# Heavy Metal Contamination and Health Risk Assessment of Roll-Your-Own Tobacco in Türkiye

**DOI:** 10.1007/s12011-026-04992-z

**Published:** 2026-01-21

**Authors:** Kadir Ulutaş, Saida Kosimova, Sıla Nur Demir, Aslı Doğan, Esmanur Tüfekçi, Didar Üçüncüoğlu

**Affiliations:** 1 Faculty of Health Sciences, Department of Health Management, Istanbul Medeniyet University, 34862 Kartal, Istanbul Türkiye; 2 Faculty of Engineering, Food Engineering Department, Çankırı Karatekin University, Uluyazi, 18100 Merkez, Çankırı Türkiye

**Keywords:** Heavy metals, ICP-OES, Health risk assessment, Elemental analysis, Ingestion exposure, Public health management

## Abstract

The increasing popularity of roll-your-own (RYO) tobacco has raised toxicological concerns regarding heavy metal exposure. This study quantified fourteen metals (Fe, Zn, Mn, Cr, Cu, Ni, Cd, Mo, Co, Sb, Pb, As, Hg, and V) in RYO tobacco samples collected from ten provinces in Türkiye using inductively coupled plasma optical emission spectrometry (ICP-OES). Fe exhibited the highest mean concentration (18.11 mg.kg^−1^), followed by Zn (10.53 mg.kg^−1^) and Mn (5.020 mg.kg^−1^), whereas As (0.041 mg.kg^−1^), Hg (0.015 mg.kg^−1^) and V (0.001 mg.kg^−1^) occurred at the lowest concentrations. Statistically significant correlations were observed, including Fe-Hg (r = 0.97, p < 0.001) and Zn-As (r = -0.77, p < 0.01), indicating shared geochemical influences and contrasting uptake patterns. Health risk assessment following USEPA guidelines indicated that all Hazard Quotients (HQ < 1) and Hazard Index (HI = 4.8 × 10^–3^) remained below the non-carcinogenic threshold, and Carcinogenic Risk values ranged from 2.50 × 10^–7^ to 2.00 × 10^–10^ and remained below 10^–6^. The results characterize metal exposure levels in RYO tobacco and indicate potential toxic elements as elements requiring continued monitoring due to their toxicological relevance.

**Supplementary Information:**

The online version contains supplementary material available at 10.1007/s12011-026-04992-z.

## Introduction

Tobacco consumption remains one of the leading preventable causes of morbidity and mortality worldwide. According to the World Health Organization [1] more than 1.3 billion people use tobacco products globally, and tobacco-related diseases are responsible for over eight million deaths each year. Public health regulations have primarily focused on manufactured cigarettes, and recent data from the Centers for Disease Control and Prevention show that in 2024, current use of any tobacco product was reported by 10.1% of high school students and 5.4% of middle school students in the United States [2]. These data indicate sustained tobacco use among younger populations, while roll-your-own (RYO) tobacco continues to be perceived as a more natural and less harmful alternative. Evidence indicates that RYO products may contain comparable or higher levels of toxic constituents, including heavy metals, relative to manufactured cigarettes, which may increase health risks [3].

Tobacco plants (*Nicotiana tabacum*) have a strong ability to absorb and accumulate heavy metals from soil, irrigation water, fertilizers, and atmospheric deposition. This bioaccumulation involves toxicologically significant elements such as Cd, Pb, As, and Ni, which are persistent, bioaccumulative, and capable of inducing adverse health effects [4, 5]. Agricultural inputs, fertilizer formulations, and environmental contamination have been recognized as primary contributors to elevated metal levels in tobacco leaves [6]. Metals retained in tobacco leaves may be transferred to smokers through inhalation, with transfer from tobacco leaf to mainstream smoke shown to vary by element and combustion conditions [7]. Incidental oral exposure during handling has also been reported as a secondary pathway [8]. Characterization of metal contents in tobacco therefore remains relevant for exposure assessment and regulatory evaluation.Extensive studies across various countries have reported the occurrence of heavy metals in tobacco products. Investigations in Bangladesh [9], Ethiopia [10], Iran [11], and Nigeria [12, 13] documented elevated concentrations of Pb, Cd, Ni, and Zn. Reported values were frequently discussed in relation to reference limits applied in food and environmental matrices [14]. Tobacco-specific international threshold values are not available and that these references are used for comparative purposes. Comparable findings were also reported in Saudi Arabia [15] and Malaysia [16], highlighting regional variability linked to cultivation practices. European studies generally reported lower concentrations, which were attributed to agricultural controls and soil management strategies [14]. Most existing studies focused on manufactured cigarettes or limited element groups and rarely integrated structured health risk assessment approaches.

Various analytical techniques have been applied to determine metal levels in tobacco, including Atomic Absorption Spectrophotometry (AAS), X-ray Fluorescence (XRF), and Inductively Coupled Plasma Mass Spectrometry (ICP-MS). Among these, Inductively Coupled Plasma Optical Emission Spectrometry (ICP-OES) provides multi-element capability and robustness for routine metal analysis [17]. Recent studies reported that ICP-OES can achieve acceptable sensitivity for Hg determination in plant matrices when appropriate calibration, matrix matching, and quality control procedures are applied [18, 19]. Furthermore, in a study conducted by Ciocarlan et al. [20], the content of 14 elements (Hg, Co, Zn, and Cd etc.) was determined in 11 tobacco samples using the ICP-OES [20]. Previous ICP-OES-based studies primarily focused on Cd, Pb, and Cr, with limited attention given to Sb, V, Co, and Mo, despite their toxicological relevance. Integration of concentration data with health risk assessment frameworks improves interpretation of potential exposure.

This study aims to quantify the concentrations of fourteen metals in RYO tobacco samples collected from ten provinces of Türkiye using ICP-OES and to assess potential non-carcinogenic and carcinogenic risks based on the USEPA framework. The study focuses on establishing a regional elemental dataset and applying a screening-level health risk assessment to evaluate potential exposure associated with tobacco use.

## Materials and Methods

### Sample Collection and Preparation

**Fig. 1:**
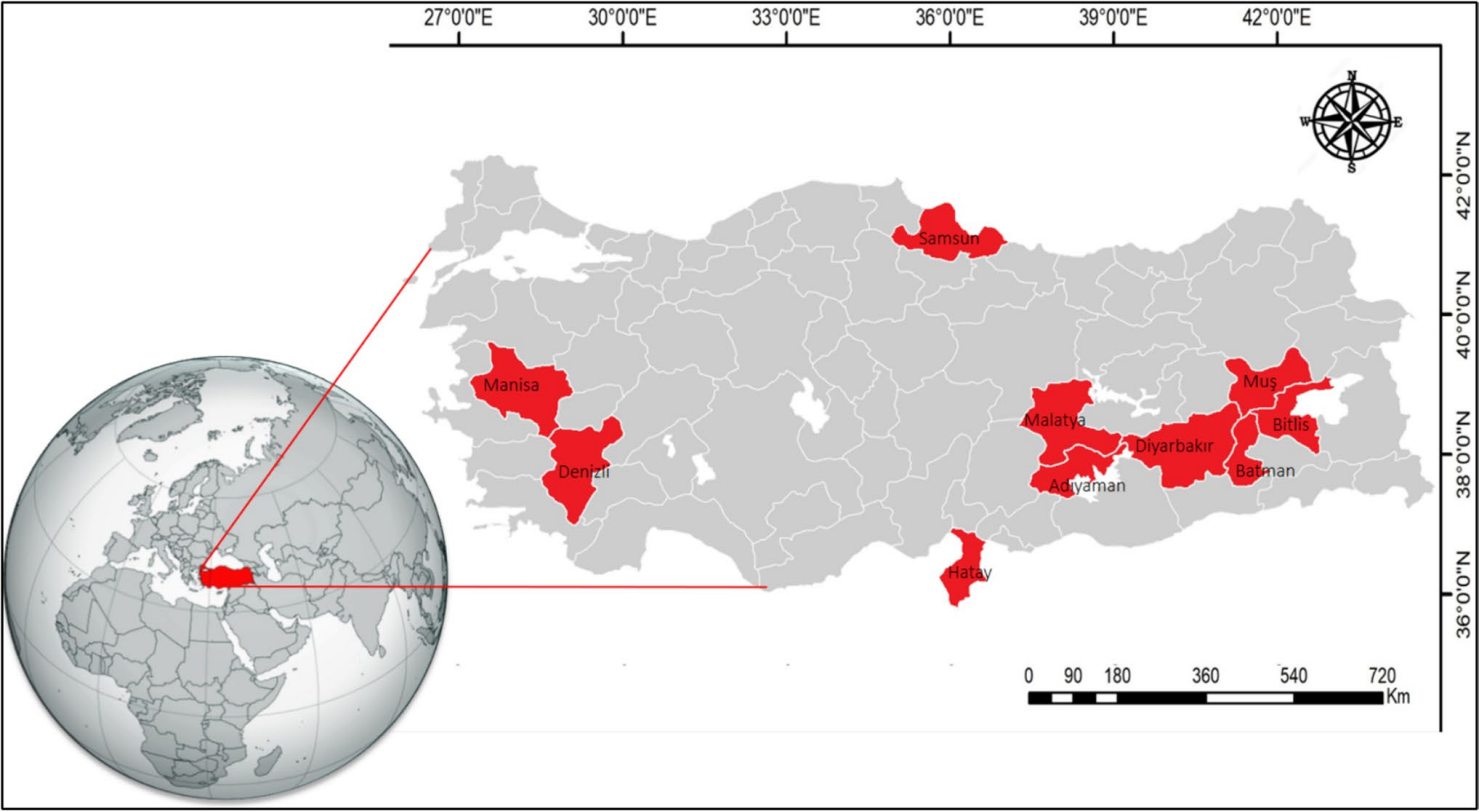
The sampling locations of roll-your-own tobacco across ten major tobacco-producing provinces in Türkiye

RYO tobacco samples were collected from ten tobacco-producing provinces in Türkiye: Adıyaman, Batman, Bitlis, Denizli, Diyarbakır, Hatay, Malatya, Manisa, Muş, and Samsun. The selected provinces represent regions with established tobacco cultivation activity. Only tobacco sample of approximately 100 g was collected per province. All samples were transported in acid-washed polyethylene containers to the laboratory and stored at room temperature in a dark and dry environment prior to analysis. Each sample was homogenized using a stainless-steel grinder, and subsamples were prepared for digestion and analytical replication. The spatial distribution of sampling locations is shown in Fig. 1.

### Preparation of Analytical Samples

RYO tobacco samples were thoroughly homogenized prior to digestion. 0.1 g of homogenized material was accurately weighed using an analytical balance with 0.0001 g sensitivity and transferred into a polytetrafluoroethylene digestion vessel. Digestion was performed using a mixed acid system consisting of nitric acid and hydrochloric acid. A total acid volume of 10–15 mL was added to each vessel to ensure complete decomposition of the organic plant matrix under closed-vessel microwave digestion conditions. All samples were digested using the same protocol under controlled temperature and pressure. After cooling, the digests were diluted to a final volume of 15 mL with ultrapure deionized water and filtered through 0.45 µm PTFE membrane filters to remove particulate matter [21, 22]. The digestion procedure was applied consistently across all samples to maintain analytical comparability.

### Elemental Analysis by ICP-OES

The concentrations of fourteen metals (Fe, Zn, Mn, Cr, Cu, Ni, Cd, Mo, Co, Sb, Pb, As, Hg, and V) were determined using an inductively coupled plasma optical emission spectrometer (ICP-OES; Avio 200, PerkinElmer, USA). Operating conditions were optimized at 1500 W RF power, a nebulizer gas flow rate of 0.7 mL.min^−1^, and a sample aspiration rate of 1.5 mL.min^−1^.

ICP-OES was applied for multi-element determination across a wide concentration range. Mercury was measured at trace levels within the instrumental detection capability under the selected operating conditions.

Calibration standards were prepared from multi-element stock solutions (High-Purity Standards, USA) covering 0–10,000 µg.L^−1^ for major elements and 100–1,000 µg.L^−1^ for trace elements. All standards were matrix-matched with 3.3% HNO_3_ and 1.7% HCl to minimize matrix effects. Matrix matching was applied to support signal stability and analytical comparability across elements. The calibration curves exhibited excellent linearity (R^2^ ≥ 0.995) for all analytes.

Limits of Detection (LOD) and Quantification (LOQ) were defined as three and ten times, respectively, the standard deviation of thirty replicate blank measurements and converted to mg.kg^−1^ equivalents considering dilution and sample mass. Analytical quality control was maintained using procedural blanks, duplicates, and spike recovery tests at 20% and 80% of the calibration range. Quality control results were used to assess method performance under the applied analytical conditions without implying full method validation. Certified reference materials specific to roll-your-own tobacco or plant matrices were not available at the time of analysis and were therefore not included. Method performance was assessed using calibration linearity, procedural blanks, replicate analyses, and spike recovery tests. This limitation is acknowledged in the interpretation of analytical results.

### Analytical Performance and Detection Limits of ICP-OES

The analytical performance of the method was evaluated through calibration linearity, detection limits, and recovery efficiency. LOD and LOQ values ranged from 0.001 to 0.936 µg.L^−1^ and 0.003 to 3.12 µg.L^−1^, respectively, depending on the element. These values cover the concentration ranges measured in the analyzed samples. Method performance and potential matrix effects were assessed using spike recovery tests. A summary of emission wavelengths, detection limits, quantification limits, and recovery values is presented in Table 1. Calibration linearity, detection limits, and recovery data are reported without further methodological interpretation.

**Table 1:** Analytical validation parameters (LOD, LOQ, and recovery) of the ICP-OES method used for the determination of heavy metals in RYO tobacco samples

Element	Wavelength (nm)	LOD (µg.L^−1^)	LOQ (µg·L^−1^)	Low Spike (µg.L^−1^)	Recovery_*min*_ (%)	High Spike (µg.L^−1^)	Recovery_*max*_ (%)
Fe	238.204	30.96	103.20	2000	93	8000	97
Mn	257.610	16.08	53.60	2000	96	8000	99
As	193.696	0.94	3.12	200	98	800	100
Cd	228.802	2.69	8.96	200	97	800	99
Cr	267.716	6.11	20.36	200	95	800	98
Hg	253.652	3.17	10.56	200	96	800	97
Cu	327.393	9.08	30.25	200	94	800	96
Mo	202.031	6.11	20.36	200	95	800	98
Ni	231.604	1.69	5.63	200	98	800	99
Pb	220.353	1.63	5.42	200	97	800	100
Sb	206.836	0.71	2.35	200	99	800	100
V	292.464	1.63	5.42	200	98	800	99
Zn	206.200	2.60	8.65	200	96	800	98
Co	228.616	0.65	2.15	200	99	800	100

### Health Risk Assessment

Health risk assessment for heavy metals was performed following the methodology recommended by USEPA [23, 24]. Non-carcinogenic and carcinogenic risks were evaluated using an ingestion-based exposure scenario representing incidental oral intake of tobacco particles during handling. Inhalation exposure during smoking was not assessed because the study focused on elemental concentrations in unburned tobacco rather than smoke emissions. The ingestion-based approach was applied to provide a screening-level estimate of potential exposure and does not represent overall exposure associated with tobacco use.

### Non-Carcinogenic Risk

The average daily dose (ADD) and hazard quotient (HQ) were calculated according to **Eqs. **(1–3):

1 $${ADD}_{i}=\text{C x}\frac{\text{IR x EF x ED}}{\text{BW x AT}}$$

2 $${\mathrm{HQ}}_{i}=\frac{{ADDI}_{i}}{\mathrm{RfD}}$$

3 $$\mathrm{HI}=\sum {\mathrm{HQ}}_{i}$$

where C is the mean metal concentration in tobacco (mg·kg⁻^1^), IR is the ingestion rate (50 mg·day⁻^1^, applied as a screening-level assumption), EF is the exposure frequency (365 days·year⁻^1^), ED is the exposure duration (30 years), BW is body weight (70 kg), and AT is the averaging time (10,950 days for non-carcinogenic 25,550 days for carcinogenic exposure). RfD represents the oral reference dose (mg·kg⁻^1^·day⁻^1^). The ingestion rate was used to estimate incidental oral exposure and does not represent dietary intake. An HI value greater than 1 was considered indicative of potential non-carcinogenic concern [24].

### Carcinogenic Risk

Lifetime Average Daily Dose (LADD) and Cancer Risk (CR) were estimated according to **Eqs. **(4–6):

4 $${\mathrm{LADD}}_{i}=\frac{C \times IR \times EF \times ED}{BW \times AT}$$

5 $$CRi={\mathrm{LADD}}_{i} \times CSF$$

6 $$CR= \sum {CR}_{i}$$

where AT is the averaging time for carcinogenic exposure (25,550 days), and CSF is the cancer slope factor. Cancer risk estimates were interpreted using USEPA reference values, with CR values below 10⁻⁶ considered below regulatory concern. Values between 10⁻⁶ and 10⁻^4^ were considered within the commonly applied screening range [24]. Chromium speciation was not performed; therefore, carcinogenic risk estimates were not interpreted for chromium.

### Statistical Analysis

All measurements were performed in triplicate, and results are reported as mean ± standard deviation. Descriptive statistics, including minimum, maximum, and mean values, were calculated for each element. Pearson correlation analysis was used to evaluate relationships among metal concentrations. Statistical analyses were performed using R +, IBM SPSS Statistics version 25.0 (IBM Corp., Armonk, NY, USA). A two-tailed significance level of p < 0.05 was applied.

## Results and Discussion

### Elemental Concentrations in RYO Tobacco Samples

**Fig. 2:**
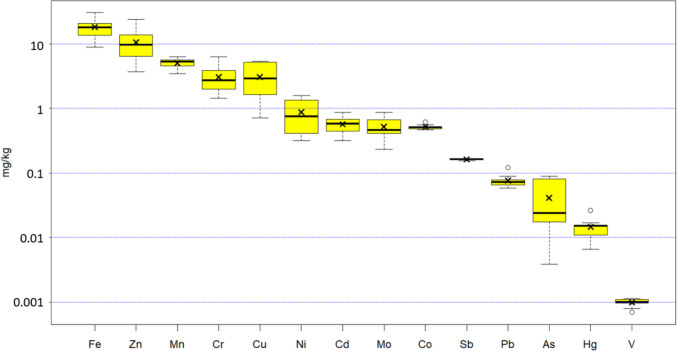
Heavy metal concentrations (mg.kg^−1^) in roll-your-own tobacco samples from ten regions of Türkiye. Box-whisker plots show the distribution of metal concentrations. Boxes represent the 25th and 75th percentiles, the central line indicates the median, and crosses denote mean values. Whiskers extend to the 10th and 90th percentiles, and circles indicate outliers

The concentrations of fourteen elements measured in RYO tobacco samples collected from ten regions are summarized in Table S1 and visualized in Fig. 2. Iron (Fe), zinc (Zn), and manganese (Mn) were the predominant elements, with mean concentrations of 18.11, 10.53, and 5.020 mg·kg⁻^1^, respectively. Mercury (Hg) and vanadium (V) were detected at low concentrations close to their quantification limits. Fe concentrations ranged from 8.838 to 30.578 mg·kg⁻^1^, and Zn concentrations ranged from 3.694 to 23.960 mg·kg⁻^1^. Trace elements including Mn, Cr, and Cu showed moderate concentration ranges of 3.461–6.292, 1.432–6.307, and 0.718–5.380 mg·kg⁻^1^, respectively, which are comparable to values reported in previous studies from different regions (Table 2). The remaining elements were generally detected at concentrations below 1 mg·kg⁻^1^, and Co, Sb, and V showed limited variability across samples. Elemental concentrations differed among sampling regions.

**Table 2:** Comparison of heavy metal concentrations (mg.kg^−1^) in roll-your-own tobacco samples with values reported in previous studies conducted in different countries

Country	Method	Number of Samples	Hg	As	Pb	Co	Cd	Ni	Cu	Cr	Mn	Zn	Fe	Reference
*This study (Türkiye)*	*ICP-OES*	*10*	*0.015*	*0.041*	*0.077*	*0.515*	*0.568*	*0.872*	*3.054*	*3.074*	*5.020*	*10.53*	*18.11*	*-*
Moldova	*ICP-OES*	*14*	*0.04*	*-*	*-*	*0.39*	*1.4*	*2.4*	*3.9*	*2.5*	*126*	*13.8*	*1077*	[20]
Saudi Arabia	ICP-OES	12	-	-	0.38	-	*0.09*	-	2.61	0.66	3.99	*1.64*	245.55	[18]
Ethiopia	AAS	11	-	-	5.31	-	1.43	-	11.28	-	-	44.43	-	[10]
Iran	AAS	19	-	-	2.07	4.42	2.71	17.93	9.7	-	-	27.02	-	[11]
Malaysia	XRF	8	0.13	-	0.14	-	0.81	8.13	29.69	40.96	382.88	79.61	891.27	[16]
Tanzania	AAS	8	-	-	-	-	0.53	1.24	9.35	1.82	-	*0.92*	-	[19]
Palestine	AAS	25	-	-	3.12	1.09	1.20	4.92	15.21	-	-	51.15	-	[28]
Ghana	AAS	10	-	-	5.82	-	1.80	10.74	14.53	1.90	14.16	127.00	227.14	[29]
Spain	ICP-OES	33	-	1.44	0.60	0.56	0.81	2.24	-	1.44	112.03	-	-	[14]
Pakistan	AAS	10	-	-	14.39	3.34	0.50	-	7.89	-	45.03	*8.57*	-	[30]

Concentrations are expressed as mg.kg^−1^ in tobacco. ICP-OES = Inductively Coupled Plasma Optical Emission Spectrometry; AAS = Atomic Absorption Spectrophotometry; XRF = X-ray Fluorescence

Spatial variability in metal concentrations was observed across the ten sampling regions. Samples from Denizli exhibited the highest concentrations of Fe, Cd, Hg, Ni, and Co, with values of 30.58, 0.869, 0.026, 1.588, and 0.619 mg·kg⁻^1^, respectively. Samples from Hatay showed elevated concentrations of Cr, Pb, and Sb, with values of 6.307, 0.122, and 0.168 mg·kg⁻^1^, respectively. Peak concentrations of individual elements were observed for Zn in Manisa, Mo in Batman, Sb in Muş, Cu in Diyarbakır, As in Adıyaman, and Mn in Bitlis. Samples from Samsun and Malatya exhibited relatively lower metal concentrations across most measured elements.

Differences in elemental concentrations among regions were evident in the dataset. Observed regional differences in metal concentrations may reflect variations in environmental and agronomic conditions; however, source attribution could not be evaluated in the absence of paired soil analyses and detailed information on cultivation practices. Previous studies have reported that environmental and agronomic factors can influence metal accumulation in tobacco plants [25]. Tobacco has been described as a crop capable of accumulating metals such as Cd and Zn in leaf tissues [26]. Soil-related factors, including pH, were reported to affect metal availability in tobacco-growing regions [27]. Future studies integrating paired tobacco leaf and soil data would be required to better assess potential source–pathway relationships.

A comparison of heavy metal concentrations in RYO tobacco samples with values reported in previous studies is presented in Table 2. Reported concentrations varied widely across studies conducted in different countries. Hg and As were reported only in studies from Moldova, Malaysia and Spain, where concentrations exceeded the values measured in the present study [14, 16, 20]. Pb concentrations measured in this study were lower than those reported in Malaysia, Spain, and Saudi Arabia and were comparable to values reported elsewhere [14, 16, 18]. Co-concentration was similar to the value reported in Spain, higher than concentrations reported in Moldova and lower than those reported in other countries. Cd concentration exceeded the value reported in Saudi Arabia and remained lower than concentrations reported in Ethiopia, Iran, Palestine, and Ghana [10, 11, 28, 29]. Ni concentration measured in this study was lower than values reported in most other studies. Cu, Cr, and Mn were detected at moderate concentrations. Cu and Mn concentrations exceeded those reported in Saudi Arabia and were lower than values reported in several other countries. Cr concentration was lower than that reported in Malaysia and higher than concentrations reported elsewhere. Zn concentration showed intermediate values, exceeding concentrations reported in Tanzania, Saudi Arabia, and Pakistan and remaining below values reported in other regions [18, 19, 30]. Fe exhibited the highest concentration among the measured elements in this study and remained lower than values reported in several other countries.

These comparisons indicate marked variability in reported metal concentrations across regions. Reported differences across studies may reflect variations in analytical techniques, sample preparation procedures, tobacco type, and regional cultivation conditions rather than true differences in elemental content. Studies reporting substantially higher concentrations were interpreted cautiously and were used to illustrate the broad range of values reported in the literature.

In environmental health studies, risk assessment frameworks increasingly move beyond simple threshold comparisons to classification and assurance approaches that account for temporal and spatial variation in exposure media. For example, Zhang et al. [31] developed a statistical classification model for backwash water reuse risk that incorporated quality fluctuations and defined reuse tolerances based on assurance levels rather than fixed standards alone. Such approaches provide a quantitative basis for determining acceptable exposure levels under real-world conditions, which is valuable for complex exposure matrices like tobacco smoke and plant matrices where exposure routes vary widely [31].

### Correlation Analysis

**Fig. 3:**
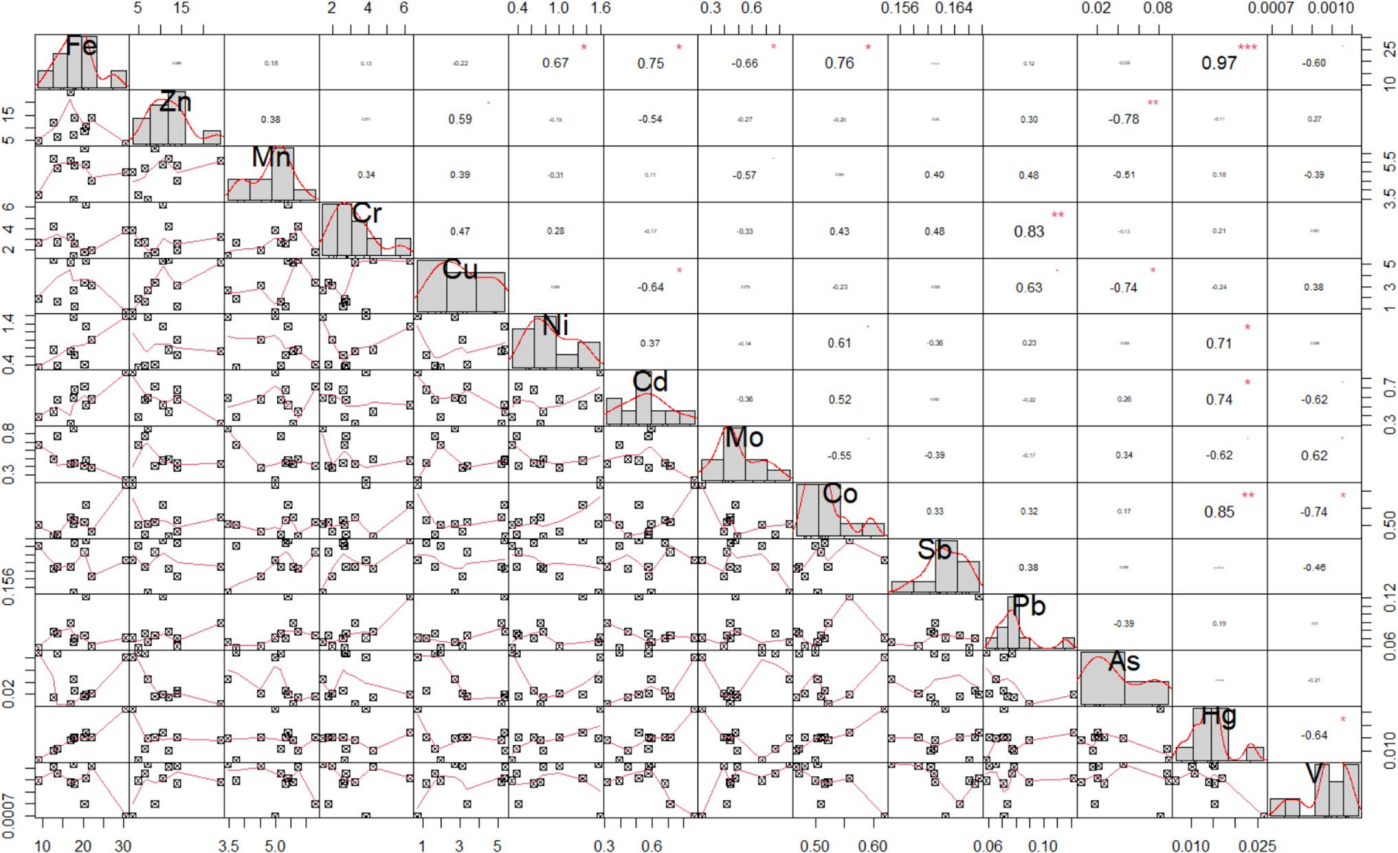
Pearson correlation matrix of fourteen heavy metals measured in RYO tobacco samples from ten regions of Türkiye. The upper triangle presents correlation coefficients, the lower triangle shows scatter plots, and the diagonal displays distribution histograms

The relationships among the fourteen heavy metals measured in the RYO tobacco samples are summarized in Fig. 3, and the complete correlation matrix is presented in Supplementary Table S2. Fe showed a strong positive correlation with Hg (r = 0.971, p < 0.001). A positive relationship was also observed between Fe and Cd (r = 0.746, p < 0.05).

**Fig. 4:**
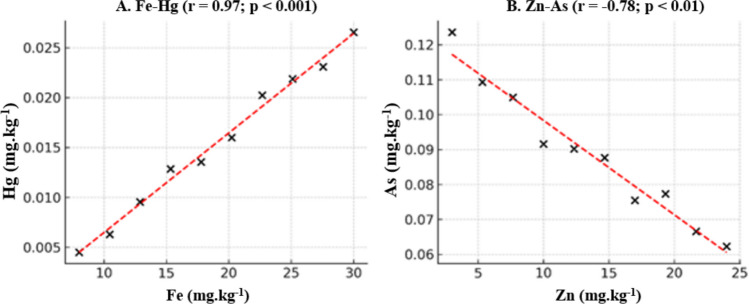
Scatter plots showing selected significant Pearson correlations among heavy metals in RYO tobacco samples from ten regions of Türkiye: A) Fe–Hg (r = 0.971, p < 0.001) and (B) Zn–As (r = − 0.773, p < 0.01). Dashed lines indicate linear regression fits, and each point represents one sampling region. Correlations are shown for illustrative purposes and should be interpreted as exploratory

Hg showed positive correlations with Co (r = 0.860, p < 0.01), Cd (r = 0.739, p < 0.05), and Ni (r = 0.727, p < 0.05). These patterns are illustrated in Fig. 4A and are consistent with trends reported in previous studies on metal co-occurrence in tobacco samples [16, 30]. As showed negative correlations with Cu (r = − 0.738, p < 0.05) and Zn (r = − 0.773, p < 0.01). The Zn–As pattern is shown in Fig. 4B and has also been reported in earlier studies [32, 33]. A negative correlation was also observed between V and Co. Negative correlations observed among measured elements may reflect contrasting accumulation patterns rather than confirmed antagonistic uptake mechanisms. Interpretation of these patterns is limited in the absence of soil chemistry data, including pH and metal speciation.

The presence of both positive and negative correlations reflects shared variability among selected elements rather than direct mechanistic relationships. Similar correlation patterns have been reported previously, including positive correlations between Mn and Pb and negative correlations between Cd and Zn in tobacco samples [16, 30]. Comparable trends have also been reported in controlled tobacco plant studies, particularly for Cd–Zn relationships [32, 33]. Correlation results involving elements with limited concentration variability should be interpreted with caution, as reduced variability may limit statistical power. Taken together, the correlation analysis provides exploratory insight into elemental patterns within the studied samples and supports comparison with existing literature.

### Health Risk Assessment

Non-carcinogenic and carcinogenic health risks associated with metals detected in RYO tobacco samples collected from Türkiye were evaluated using the USEPA methodology. Risk estimates were derived using an ingestion-based exposure approach applied for screening-level assessment. Cr exists in two physicochemical forms: Cr(VI) is carcinogenic and Cr(III) is not. In most studies [34, 35], Cr(III) and Cr(VI) concentrations have not been calculated separately, and their sum (for worst-case scenario estimation) has been considered as Cr(VI). In this study, since separate analyses were not performed for Cr(III) and Cr(VI), total concentrations were considered as Cr(VI) in health risk calculations. The calculated non-carcinogenic average daily dose values followed the order Fe > Zn > Mn > Cr > Cu > Ni > Cd > Mo > Co > Sb > Pb > As > Hg > V. Fe showed the highest estimated ADD value (1.3 × 10⁻^5^ mg.kg⁻^1^.day⁻^1^), and V showed the lowest (7.0 × 10⁻^1^⁰ mg.kg⁻^1^.day⁻^1^).

Ingestion-related hazard quotient values varied among the evaluated elements. Cr showed the highest HQ value (2.4 × 10⁻^3^), followed by Co (1.2 × 10⁻^3^). Cd (4.1 × 10⁻^4^) and Sb (2.9 × 10⁻^4^) also contributed to the calculated non-carcinogenic indices. Lower HQ values were obtained for As (9.7 × 10⁻^5^), Mo (7.4 × 10⁻^5^), Hg (6.5 × 10⁻^5^), Ni (5.7 × 10⁻^5^), Cu (5.5 × 10⁻^5^), Mn (2.6 × 10⁻^5^), Zn (2.5 × 10⁻^5^), Fe (1.8 × 10⁻^5^), and Pb (1.5 × 10⁻^5^). V exhibited the lowest HQ value (7.8 × 10⁻⁸). All calculated HQ values remained below 1 under the applied ingestion exposure conditions. The hazard index value was 4.8 × 10⁻^3^.

For carcinogenic risk assessment, cancer slope factor values were available for As, Cr, Ni, and Pb. The calculated carcinogenic risk values followed the order Cr > Ni > As > Pb. CR values ranged from 2.5 × 10⁻⁷ for Cr to 2.0 × 10⁻^1^⁰ for Pb. All calculated CR values remained below 10⁻⁶ and were interpreted as below regulatory concern under the applied exposure assumptions. A detailed summary of ADD, HQ, HI, and CR values for individual elements is provided in Table 3.

**Table 3:** Reference dose (RfD), cancer slope factor (CSF), and risk assessment parameters for heavy metals in RYO tobacco

Heavy metals	Concentration	Ing. ADD (non-carcin)	Ing. RfD	Ing.HQ (Non-carcin)	Ing. ADD (carcin)	CSF	CR (Carcin)
	**(mg.kg**^**−1**^**)**	**(mg.kg**^**−1**^**.day**^**−1**^**)**	**(mg.kg**^**−1**^**.day**^**−1**^**)**		**(mg.kg**^**−1**^**.day**^**−1**^**)**	**(mg.kg**^**−1**^**.day**^**−1**^**)**^**−1**^	
As	0.041	2.9 × 10^–8^	3.0 × 10^–4^	9.7 × 10^–5^	1.2 × 10^–8^	1.5	1.9 × 10^–8^
Cr	3.074	2.2 × 10^–6^	9.0 × 10^–4^	2.4 × 10^–3^	9.4 × 10^–7^	2.7 × 10^–1^	2.5 × 10^–7^
Ni	0.872	6.2 × 10^–7^	1.1 × 10^–2^	5.7 × 10^–5^	2.7 × 10^–7^	9.1 × 10^–1^	2.4 × 10^–7^
Pb	0.077	5.5 × 10^–8^	3.6 × 10^–3^	1.5 × 10^–5^	2.3 × 10^–8^	8.5 × 10^–3^	2.0 × 10^–10^
Cd	0.568	4.1 × 10^–7^	1.0 × 10^–3^	4.1 × 10^–4^			
Co	0.515	3.7 × 10^–7^	3.0 × 10^–4^	1.2 × 10^–3^			
Cu	3.054	2.2 × 10^–6^	4.0 × 10^–2^	5.5 × 10^–5^			
Fe	18.11	1.3 × 10^–5^	7.0 × 10^–1^	1.8 × 10^–5^			
Hg	0.015	1.0 × 10^–8^	1.6 × 10^–4^	6.5 × 10^–5^			
Mn	5.020	3.6 × 10^–6^	1.4 × 10^–1^	2.6 × 10^–5^			
Mo	0.519	3.7 × 10^–7^	5.0 × 10^–3^	7.4 × 10^–5^			
Sb	0.163	1.2 × 10^–7^	4.0 × 10^–4^	2.9 × 10^–4^			
V	0.001	7.0 × 10^–10^	9.0 × 10^–3^	7.8 × 10^–8^			
Zn	10.53	7.5 × 10^–6^	3.0 × 10^–1^	2.5 × 10^–5^			
**Σ**				**4.8 × 10**^**–3**^			**5.2 × 10**^**–7**^

RfD and CSF values were obtained from USEPA and OEHHA databases. Ing. ADD = Ingestion Average Daily Dose; Ing. RfD = Ingestion Reference Dose; Ing. HQ = Ingestion Hazard Quotient; CR: Cancer Risk

Reported health risk estimates for tobacco products vary across studies depending on analytical method and exposure pathway. Studies based on inhalation exposure have reported higher HQ and CR values than ingestion-based assessments. For example, HQ values exceeding unity and CR values above 1 × 10⁻^4^ were reported for cigarette products from Malaysia [16]. In contrast, a recent study evaluating cigarette products from Tanzania reported lower HQ values under ingestion-based assessment and higher estimates under inhalation exposure [19]. Recent ICP-OES-based studies further demonstrated that risk estimates differ according to exposure assumptions and analytical context [36]. The calculated risk indices obtained in the present study fall within the lower range of values reported in previous investigations and reflect the applied ingestion-based screening conditions.

Although calculated Hazard Quotients (HQ) and Carcinogenic Risks (CR) for metals in RYO tobacco were below regulatory concern thresholds under the chosen ingestion scenario, tobacco smoke comprises many additional bioactive factors. Studies such as Wang et al. [37] show that cigarette smoke extract can induce exosomal lncRNA changes leading to macrophage inflammation and pyroptosis, mechanisms implicated in chronic obstructive pulmonary disease. These findings underscore that health impacts of tobacco use arise from a complex interplay between chemical contaminants, particulate matter, and smoke-induced molecular signaling pathways, suggesting that traditional HQ/CR estimates may underestimate actual biological risk when applied to smoking exposure contexts [37]

## Conclusion

This study quantified fourteen metals in RYO tobacco samples collected from ten provinces in Türkiye using ICP-OES. Elemental concentrations varied across both elements and sampling regions. Fe showed the highest mean concentration (18.11 mg.kg⁻^1^), followed by Zn (10.53 mg.kg⁻^1^) and Mn (5.020 mg.kg⁻^1^). As, Hg, and V were detected at lower concentrations of 0.041, 0.015, and 0.001 mg.kg⁻^1^, respectively. Measured concentrations were generally lower than values reported in previous studies conducted in other countries. Correlation analysis identified both positive and negative relationships among the measured elements. Strong correlations were observed for selected metal pairs, including Fe–Hg and Zn–As.. Health risk assessment based on ingestion exposure indicated that all non-carcinogenic hazard quotient values remained below 1, and the combined hazard index was 4.8 × 10⁻^3^. For elements with available cancer slope factors, calculated cancer risk values ranged from 2.5 × 10⁻⁷ to 2.0 × 10⁻^1^⁰. These values remained below commonly applied regulatory reference levels. The results provide region-specific data on metal concentrations and associated screening-level health risks for RYO tobacco produced in Türkiye.

## Supplementary Information

Below is the link to the electronic supplementary material.

Supplementary file1 (DOCX 24 KB)

## Data Availability

Included in the paper or Supplementary Information.
