# Supplementary material for: Heavy Metal Contamination and Health Risk Assessment of Roll-Your-Own Tobacco in Türkiye
Source: Biol Trace Elem Res. 2026 Jan 21;204(7):4745–54. doi: 10.1007/s12011-026-04992-z (PMC13319628; doi:10.1007/s12011-026-04992-z)
Supplement: Supplementary file 1 — Supplementary file1 (DOCX 24 KB) [file 12011_2026_4992_MOESM1_ESM.docx]

**Heavy Metal Contamination and Health Risk Assessment of Roll-Your-Own Tobacco in Türkiye**

**Supplementary Table S1.** Mean, median, minimum, maximum, and standard deviation (SD) of heavy-metal concentrations (mg.kg^-1^) in roll-your-own tobacco samples from ten regions of Türkiye

| **Element** | **Mean** | **Median** | **Min** | **Max** | **SD** |
| --- | --- | --- | --- | --- | --- |
| Fe | 18.11 | 17.76 | 8.848 | 30.58 | 5.975 |
| Mn | 5.020 | 5.247 | 3.461 | 6.292 | 0.884 |
| Zn | 10.53 | 9.710 | 3.694 | 23.96 | 5.909 |
| Cr | 3.074 | 2.691 | 1.432 | 6.307 | 1.437 |
| Cu | 3.054 | 2.896 | 0.718 | 5.380 | 1.746 |
| Ni | 0.872 | 0.744 | 0.319 | 1.588 | 0.482 |
| Pb | 0.077 | 0.073 | 0.058 | 0.122 | 0.018 |
| Cd | 0.568 | 0.576 | 0.314 | 0.869 | 0.163 |
| As | 0.041 | 0.024 | 0.004 | 0.089 | 0.033 |
| Hg | 0.015 | 0.015 | 0.0007 | 0.026 | 0.005 |
| Sb | 0.163 | 0.162 | 0.155 | 0.168 | 0.004 |
| V | 0.0010 | 0.0010 | 0.0007 | 0.0011 | 0.0001 |
| Mo | 0.519 | 0.460 | 0.233 | 0.869 | 0.192 |
| Co | 0.515 | 0.507 | 0.468 | 0.619 | 0.045 |

**Supplementary Table S2.** Detailed Pearson correlation coefficients (r) and significance levels (p) among selected heavy metals in roll-your-own tobacco samples

| **Variable** | **Fe** | **Mn** | **As** | **Cd** | **Cr** | **Hg** | **Cu** | **Mo** | **Ni** | **Pb** | **Sb** | **V** | **Zn** | **Co** |
| --- | --- | --- | --- | --- | --- | --- | --- | --- | --- | --- | --- | --- | --- | --- |
| **Fe** | 1 | *0.615* | *0.805* | *0.013* | *0.720* | *0.000* | *0.542* | *0.036* | *0.033* | *0.721* | *0.960* | *0.070* | *0.807* | *0.010* |
| **Mn** | 0.182 | 1 | *0.130* | *0.767* | *0.333* | *0.684* | *0.266* | *0.087* | *0.383* | *0.165* | *0.259* | *0.275* | *0.279* | *0.856* |
| **As** | -0.090 | -0.513 | 1 | *0.476* | *0.721* | *0.987* | *0.015* | *0.329* | *0.884* | *0.261* | *0.937* | *0.544* | *0.009* | *0.654* |
| **Cd** | 0.746* | 0.108 | 0.256 | 1 | *0.648* | *0.015* | *0.047* | *0.313* | *0.293* | *0.567* | *0.794* | *0.057* | *0.110* | *0.124* |
| **Cr** | 0.130 | 0.342 | -0.130 | -0.165 | 1 | *0.560* | *0.172* | *0.353* | *0.432* | *0.003* | *0.170* | *0.825* | *0.846* | *0.219* |
| **Hg** | 0.971** | 0.148 | -0.006 | 0.739* | 0.210 | 1 | *0.475* | *0.068* | *0.017* | *0.598* | *0.899* | *0.049* | *0.722* | *0.001* |
| **Cu** | -0.220 | 0.389 | -0.738* | -0.638* | 0.468 | -0.256 | 1 | *0.842* | *0.944* | *0.054* | *0.846* | *0.283* | *0.072* | *0.525* |
| **Mo** | -0.665* | -0.567 | 0.345 | -0.356 | -0.329 | -0.597 | -0.073 | 1 | *0.694* | *0.650* | *0.247* | *0.058* | *0.458* | *0.097* |
| **Ni** | 0.672 | -0.310 | -0.053 | 0.369 | 0.281 | 0.727* | 0.026 | -0.143 | 1 | *0.517* | *0.361* | *0.878* | *0.678* | *0.061* |
| **Pb** | 0.130 | 0.476 | -0.393 | -0.206 | 0.826** | 0.191 | 0.623 | -0.164 | 0.233 | 1 | *0.262* | *0.980* | *0.410* | *0.379* |
| **Sb** | 0.018 | 0.394 | 0.029 | 0.095 | 0.470 | 0.046 | -0.071 | -0.404 | -0.324 | 0.393 | 1 | *0.161* | *0.854* | *0.308* |
| **V** | -0.595 | -0.383 | -0.219 | -0.618 | -0.080 | -0.634* | 0.377 | 0.616 | -0.056 | -0.009 | -0.479 | 1 | *0.446* | *0.014* |
| **Zn** | -0.089 | 0.380 | -0.773** | -0.537 | 0.071 | -0.129 | 0.591 | -0.266 | -0.150 | 0.294 | -0.067 | 0.272 | 1 | *0.588* |
| **Co** | 0.765** | 0.066 | 0.162 | 0.519 | 0.427 | 0.860** | -0.229 | -0.553 | 0.610 | 0.313 | 0.359 | -0.744* | -0.196 | 1 |

*The lower-left triangle of the table presents the Pearson correlation coefficients (r), where superscript stars indicate the level of statistical significance. The upper-right triangle contains the corresponding significance values (p), shown in italics. Correlations with p < 0.001 are classified as very highly significant, those with p < 0.01 as highly significant, and those with p < 0.05 as significant, based on two-tailed tests. Values with p > 0.05 indicate correlations that are not statistically significant.*
